# Type D Personality, Concomitant Depressive and Anxiety Disorders, and Treatment Outcomes in Somatic Symptom and Related Disorders: An Observational Longitudinal Cohort Study

**DOI:** 10.3389/fpsyt.2019.00417

**Published:** 2019-06-19

**Authors:** Lars de Vroege, Eric W. de Heer, Eva van der Thiel, Krista C. van den Broek, Jonna F. van Eck van der Sluijs, Christina M. van der Feltz-Cornelis

**Affiliations:** ^1^Department Tranzo, Tilburg School of Social and Behavioral Sciences, Tilburg University, Tilburg, Netherlands; ^2^Clinical Centre of Excellence for Body, Mind and Health, GGz Breburg, Tilburg, Netherlands; ^3^MHARG, Department of Health Sciences, Hull York Medical School, University of York, York, United Kingdom

**Keywords:** Type D personality, somatic symptom and related disorders, treatment outcome, anxiety, depression

## Abstract

**Objective:** To establish the prevalence of Type D personality in patients with somatic symptoms and related disorders and to evaluate the association of Type D personality with treatment outcomes. This study explores the effect of Type D personality and its two traits, negative affectivity (NA) and social inhibition (SI).

**Methods:** In this longitudinal observational cohort study, we assessed the prevalence of Type D in 212 patients presenting themselves at a clinic in Tilburg, the Netherlands. We explored psychological and physical treatment outcomes of a multimodal treatment tailored to patient needs in relation to Type D scores. We explored the differences with regard to physical symptoms, anxiety, and depression. We also explored the differences between patients with and without Type D personality who completed treatment with regard to the baseline scores of physical symptoms, anxiety, and depression. We explored the association between Type D personality and treatment outcome using the traditional dichotomous method and the dimensional method (with main effects of NA and SI, and the interaction of NA × SI).

**Results:** Of the 212 patients with Somatic Symptom and Related Disorders (SSRD), those with Type D personality (181: 61.8%) had experienced significantly higher levels of depression [*t* = 4.404, *p* < .001] and anxiety [*t* = 3.757, *p* < .001]. Of the 212, 187 patients completed treatment. Mean scores improved significantly for the whole patient group after treatment with regard to depression (*p* < .001), anxiety (*p* < .001), and physical symptoms (*p* < .001). At baseline, patients with Type D personality had significantly higher scores in anxiety [*F* = 15.707, *p* < .001] and depression [*F* = 19.392] than patients without Type D personality who completed treatment. After controlling for the high baseline scores with regard to physical symptoms, anxiety, or depression, only the effect of Type D personality on remission of anxiety was significant (*OR* = .33, *p* = 0.39). Neither NA and SI nor the interaction of NA × SI was associated with the treatment outcome.

**Conclusions:** This study shows that Type D personality occurs frequently in patients with SSRD. Type D personality only decreases the probability of remission of anxiety as a treatment outcome, and both NA and SI play a role in this. Type D personality did not decrease remission either of physical symptoms or of depression. Hence, both NA and SI factors may be expressions of anxiety mostly in type D.

## Introduction

### Background

The fifth edition of the *Diagnostic and Statistical Manual of Mental Disorders* (*DSM*) includes Somatic Symptom and Related Disorders (SSRD) (1), which replaces the Somatoform Disorders section of the *DSM-IV-TR* (2). The SSRD classification has as a common feature: the prominence of somatic symptoms associated with significant distress and impairment, irrespective of the question of whether the somatic symptoms co-occur with a diagnosed chronic medical condition (1). As such, SSRD has a broader scope than have the former somatoform disorders, which were exclusively linked to the concept of somatization (3) (i.e., having the tendency to experience and communicate psychological distress in the form of somatic symptoms and to seek medical help for them).

The experience of somatic symptoms in somatization has been associated with harm avoidance and negative affectivity (NA) (4). Compared to non-somatizing patients, patients with somatization show more self-defeating, depressive, and passive–aggressive personality traits and neuroticism, and less agreeableness and extraversion (5).

A personality construct that might be relevant in SSRD is Type D personality. This construct combines two traits: NA, the tendency to experience negative emotions across time and situations (6, 7); and social inhibition (SI) (6), the tendency to inhibit the expression of emotions and behaviors in social interactions to avoid disapproval (8). Individuals with high levels of both NA and SI are classified as individuals with Type D (i.e., distressed) personality (6). Previous studies showed a prevalence range of 21–33% (6, 9) of Type D personality in the general population, 28–53% (6) in the population of people with cardiac diseases or disorders, 36% in people with tinnitus (10), 43% in people with chronic pain (11), and 57% in people with fibromyalgia (12).

In the populations of people with cardiac diseases, Type D personality is associated with emotional distress, such as anxiety and depression (9, 13), poor health status and quality of life, myocardial infarcts, high mortality rates (14), high utilization of health services (9), poor self-management (13), and higher levels of anxiety and depression after cardiac rehabilitation compared to patients without Type D personality (15). An earlier study explored the influence of SI and NA separately and reported that NA is primarily associated with poorer treatment outcomes in people with fibromyalgia (12). The prevalence of Type D personality in patients with fibromyalgia was 56.5%. Furthermore, worse mental and physical health was associated with NA (12).

A systematic review focusing on other patient populations, such as patients with chronic pain and traumatic brain injuries, found an association of Type D personality with negative emotions (i.e., depression and anxiety), poor treatment adherence, and an increased number or severity of reported health symptoms (16). However, the prevalence of Type D personality in SSRD and the association with treatment outcome are unknown.

### Rationale

Taking the abovementioned into account, the prevalence of Type D personality in patients with SSRD is unknown. Furthermore, patients with SSRD and Type D personality might benefit less from treatment than would patients with SSRD who do not have Type D personality. However, to date, no published studies have investigated the prevalence of Type D personality in SSRD patients, or its association with treatment outcomes. This study aims to explore this. Because the dichotomous conceptualization of Type D personality construct has been questioned (17, 18), we also explore the effect of NA and SI both separately and combined in order to establish if one of the factors composing Type D might be more relevant to treatment outcomes.

### Objectives

1. To assess the prevalence rate of Type D personality in patients with SSRD.
2. To determine the association between Type D personality and physical and psychological treatment outcomes in patients with SSRD.
3. To explore the effect of NA and SI separately and as an interaction (NA × SI) on physical and psychological treatment outcomes.

We hypothesized a higher prevalence of Type D personality in patients suffering from SSRD compared to previous studies in other patient groups. We also hypothesized that patients with Type D personality had worse physical and psychological treatment outcomes than had patients without Type D personality because previous studies showed that Type D personality was associated with an increased experience of symptoms. In view of previous research, we hypothesized that the association between NA and treatment outcomes would be worse than the association between SI or NA × SI and treatment outcomes would be.

## Methods and Materials

### Study Design

This study used the longitudinal observational method in a clinical setting. The cohort consisted of outpatients with SSRD who were treated at the Clinical Centre of Excellence for Body, Mind, and Health (Dutch abbreviation: CLGG), a department for treatment of complex SSRD of GGz Breburg, a specialty mental health institution (SMHI) in Tilburg, the Netherlands. CLGG uses computerized Patient Routine Outcome Monitoring (PROM; assessed every 6 weeks), which consists of a set of questionnaires that give an indication of the severity and frequency of the symptom(s) (19). For this study, we used a selection of the PROM questionnaires at baseline and at the end of treatment. Consecutive patients who had been referred to CLGG between August 2013 and April 2016 were included in the study. Patients are referred to CLGG by general practitioners, by medical specialists from general hospitals, or by psychiatrists working in Psych Med units of general medical hospitals or in SMHIs. They have been suffering from somatic symptoms causing high levels of distress for an average of 8 years and 6 months and have received treatment for their condition without solace for an average of 7 years. They suffer from highly complex SSRD as established in earlier research by this group (20).

All patients were informed before intake that the PROM data pertaining to their treatment could be used on an anonymous basis for research and that they could indicate during the intake if they declined the use of their data for scientific purposes. If the patient declined, this was recorded in the administration system and the data of these patients were excluded from the study. No consent regarding the use of their data for scientific purposes did not have any consequences for treatment at our center. The study protocol was approved by the scientific committee of GGz Breburg (file number: CWO 2014-11).

### Participants

Patients of 18 years of age or older who completed the intake and baseline PROM measures were evaluated for eligibility. Patients were excluded if they were engaged in personal or professional injury procedures (e.g., work-related lawsuits), had an IQ below 80 as assessed with the Dutch Adult Reading Test (21), or were, for whatever reason, unable to follow treatment at CLGG.

### Treatment

After the intake, treatment options at CLGG were offered to the patients in a Shared Decision Making (SDM) model (19). CLGG offers a multimodal treatment that builds on treatment modes suggested in the multidisciplinary guideline for medically unexplained symptoms and somatic disorders (22, 23), such as acceptance and commitment therapy (ACT), cognitive behavioral therapy (CBT), and problem-solving treatment (PST) provided by trained and supervised psychologists sequentially, depending on patients’ preferences and needs. This was provided in combination with psychiatrist- or physician-prescribed pharmacotherapy focusing on chronic pain (24) or comorbid depressive or anxiety disorders. Every 3 months, both psychotherapeutic and pharmacotherapeutic treatment were adjusted based on progress in terms of PROM and using the SDM model with the patient (19), after multidisciplinary team consultations. A pilot study evaluating this treatment model showed high compliance among patients (19). On average, patients were treated for 1 year according to this multimodal treatment model.

### Instruments

#### Patient Characteristics

Sociodemographic variables included age, education level, and gender. Educational level was classified following Verhage (25). For this study, we dichotomized educational level due to the relatively small sample of patients who completed treatment. Educational level was categorized as follows: the five lowest classifications were classified as “low” and the two highest classifications were classified as “high.” *DSM-5* SSRD diagnoses were established by two psychiatrists after psychiatric interview.

#### Questionnaire Assessment

The standard intake procedure at the CLGG consists of a questionnaire assessment during intake (referred to as baseline measurement), a case history assessment, a physical assessment, a psychiatric evaluation, and a psycho-diagnostic assessment. The DS14 Questionnaire (DS14) (6) was self-administered during the psycho-diagnostic assessment at intake.

##### Type D Personality

Type D personality was measured at intake by means of the Type D scale 14 (DS14) (6). This self-report questionnaire consists of two seven-item subscales: one scale that assesses NA and another that assesses SI. Items were scored on a five-point Likert scale having a range of 0 (false) through 4 (true). Total scores on each of the two subscales can range from 0 to 28, with higher scores indicating higher levels of NA and/or SI. The DS14 has good psychometric properties (6). Individuals who score at least 10 on each of the subscales are classified as having a Type D personality (6). This means that the Type D personality is conceived as a dichotomous typology. The typology may be useful from a clinical perspective where dichotomous treatment decisions have to be made.

##### Physical Symptoms

The Physical Symptom Checklist (PSC) (26) is a 51-item self-report questionnaire that measures physical symptoms during the last week. The score descriptions are as follows: 0, does not burden me; 1, sometimes burdens me; 2, often burdens me; and 3, always burdens me. We followed the guidelines of Van Hemert (26), in which the item scores were converted into dichotomous scores. Scores of 0 and 1 were transformed to 0, and scores of 2 and 3 were transformed to 1. In this way, a symptom is present when rated a 2 or 3. The total score represents the number of symptoms that were present in the last week. Total scores ranged from 0 to 51. A higher score on the PSC indicates a higher number of symptoms present in the last week (26). The PSC is a valid Dutch questionnaire to assess physical symptoms (27). However, no validated cutoff scores are present. The mean score for patients visiting the general practitioner’s office equaled six for women and four for men (28). Regarding these mean scores of the PSC in a general practitioner’s sample, we defined treatment remission as a score of below 5 at the end of treatment.

##### Anxiety

To assess anxiety symptoms, the Generalized Anxiety Disorder questionnaire (GAD-7) was used. The GAD-7 is a seven-item self-report questionnaire that measures symptoms of anxiety during the last 2 weeks. For each item, scores range from 0 (not at all) to 3 (nearly every day) (29). Total scores range from 0 to 21, with higher scores indicating higher levels of anxiety symptoms. The GAD-7 is a reliable questionnaire (29, 30) and has been adapted in Dutch and well validated in the Netherlands (31, 32).

##### Depression

To assess depression, the Patient Health Questionnaire-9 (PHQ-9) (33) was used. The PHQ-9 is a nine-item self-report questionnaire. For each item, scores range from 0 (not at all) to 3 (nearly every day). Total scores range from 0 to 27, with higher scores indicating higher levels of depressive symptoms (33). The PHQ-9 has been shown to be a reliable questionnaire (33, 34) and has been adapted in Dutch and well validated in the Netherlands (34).

### Treatment Outcomes

#### Remission

For each of the outcome measures (PSC, GAD-7, and PHQ-9), remission on a single outcome was defined as having a score that dropped below 5 after treatment (35). Remission of symptoms is defined as the point after treatment at which a patient’s score that had exceeded the clinical cutoff at baseline no longer exceeds it.

#### Treatment Response

Response is defined as a reduction of the score (on the PSC, the GAD-7, or the PHQ-9) of at least 50% after the therapy compared to the score at intake, as defined similarly in earlier studies (36, 37).

### Statistical Methods

To describe patient characteristics and the prevalence of Type D personality, we obtained descriptive statistics. To test whether the Type D personality group and the non-Type D personality group differed on baseline characteristics, we executed independent *t* tests and chi-square tests. Cohen’s *d* was used to gauge the effect size. Effect sizes of about *d* = 0.2 are considered small, those of about *d* = 0.5 are medium, and those of *d* ≥ 0.8 are large (38). For the PSC, the GAD-7, and the PHQ-9, we also studied mean differences between raw scores before and after treatment. Paired-sample *t* tests were conducted to test if patients who completed treatment had, on average, significant lower physical, anxiety, and depressive symptoms at the end of treatment. Unpaired *t* tests were done for the Type D and non-Type D groups separately. Using the McNemar test, we also inspected the proportion of patients having a clinical diagnosis to see changes between intake and after the treatment. We also performed an analysis of variance (ANOVA) for all outcomes of interest with Type D personality as a between-subject factor for patients who completed treatment.

Regarding the third objective, to study the hypothesized relationship of Type D personality with the dichotomous outcome variables, we used two different analyses. The first analysis used the operationalization of Type D as described by Denollet (6). This method uses cutoff scores for the two subscales of Type D, i.e., NA and SI, and we combined them to determine a categorical classification of patients as having Type D or non-Type D. We fitted Model 1, which included the background variables of age, gender, and education level; in Model 2, we added the dichotomous Type D variable as predictor; and in Model 3, we controlled for baseline measures of the outcome measurement of interest, namely, the PSC, the GAD-7, or the PHQ-9. These results are shown in **Table 3**.

In the second analysis, we explored the extent to which NA, SI, and their interaction (NA × SI) predicted treatment outcomes. For this approach, the following three models were applied. Model 1 included the background variables of age, gender, and education level; Model 2 added the variables NA and SI (i.e., main effects only); and Model 3 added the interaction term between NA and SI, denoted NA × SI. Significant findings were controlled for the measurement of interest using the baseline measurement of the PSC, the GAD-7, or the PHQ-9 by using a model in which this baseline measurement was added. These results are shown in **Table 4**.

Likelihood ratio tests were used to see whether model fit improved when adding predictors. Nagelkerke’s pseudo *R* ^2^ was used to gauge the effect sizes. Following Nagelkerke (39), we interpreted the pseudo *R* ^2^ as the proportion of the variation explained by the model, but we are aware that pseudo *R* ^2^s are not the same as *R* ^2^s in linear models. For all models, we used Cohen (38) guidelines for the *R* ^2^s to interpret Nagelkerke’s pseudo *R* ^2^ (i.e., *R* ^2^ = .02 were considered small, *R* ^2^ = .13 were considered medium, and *R* ^2^ ≥ .26 were considered large). All analyses were performed by means of IBM SPSS statistics 22 (40).

## Results

### Sample Characteristics

**Figure 1:**
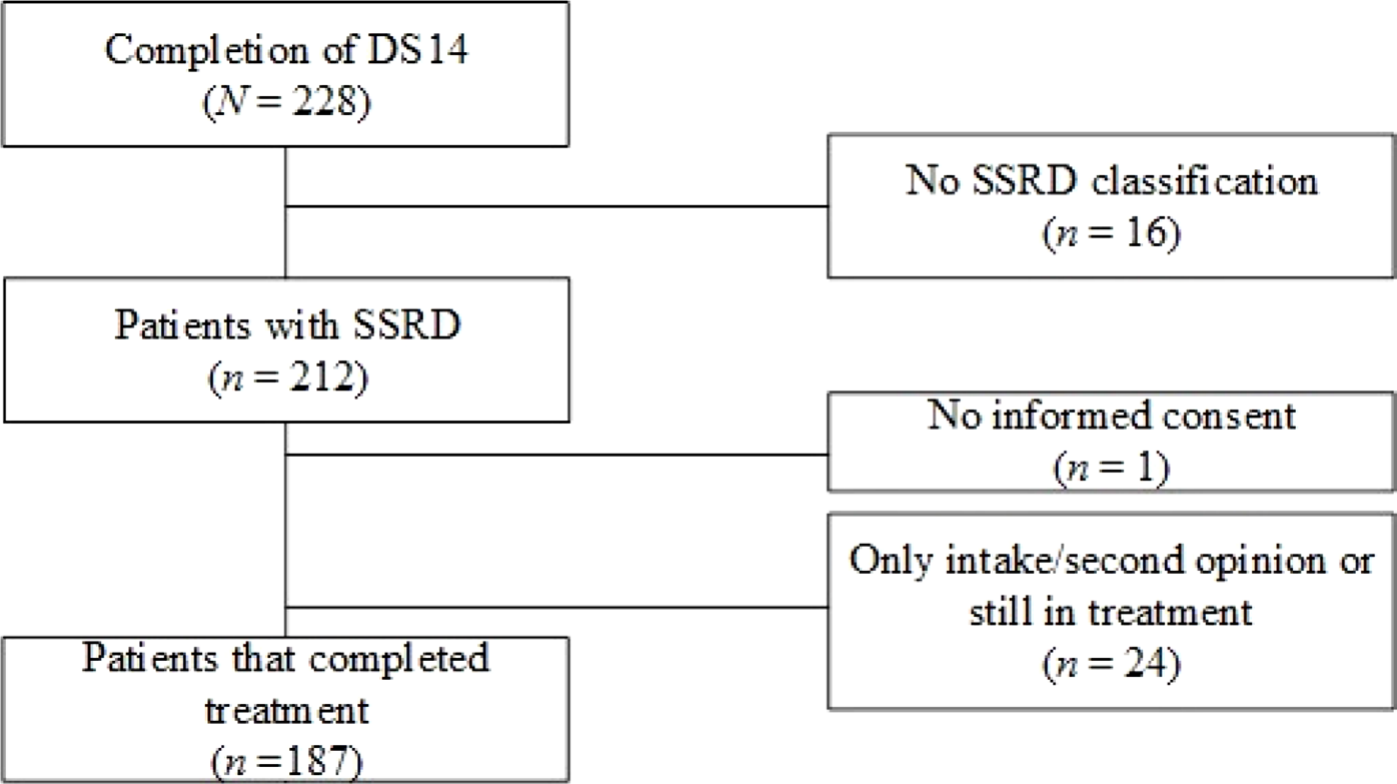
Flowchart of patients included in the study. Sample size is given for patients who completed the Treatment and Questionnaire Assessment.* Abbreviations:* DS14, Type D Scale; SSRD, Somatic Symptom and Related Disorders.

**Figure 1** displays a flowchart of the study. A total of 228 patients completed the DS14 questionnaire at baseline. Of these patients, 16 (7.0%) were not diagnosed as having SSRD and were excluded from the analyses. Of the remaining 212 patients, 187 (88.2%) patients completed treatment. **Table 1** shows the background characteristics. Of the patients who completed treatment, 15 (8.0%) were diagnosed with a conversion disorder, 11 (5.9%) with an illness anxiety disorder, and 161 (86.1%) with a somatic symptom disorder.

**Table 1:** Sociodemographic variables, predictors, and outcome variables of the total sample of patients with and without Type D personality and of patients with and without Type D personality who completed treatment, at baseline.

		Total sample (*n* = 212)				Patients who completed treatment (*n* = 187)		
	Total (*N* = 212)	Type D (*n* = 131)	Non-Type D (*n* = 81)		Total (*N* = 187)	Type D (*n* = 117)	Non-Type D (*n* = 70)	
Sociodemographic variables	*M* (*SD*)/*n* (%)	*M* (*SD*)/*n* (%)	*M* (*SD*)/*n* (%)	*p*	*M* (*SD*)/*n* (%)	*M* (*SD*)/*n* (%)	*M* (*SD*)/*n* (%)	*p*
Gender (male)	82 (38.67%)	56 (42.75%)	26 (32.10%)	.122^a^	72 (38.50%)	50 (42.74%)	22 (31.43%)	.124^a^
Age in years	42.51 (12.43)	41.26 (11.53)	44.54 (13.58)	.061^b^	42.34 (12.36)	41.15 (11.37)	44.31 (13.70)	.091^b^
Education level (low)	57 (26.89%)	36 (27.48%)	21 (25.93%)	.804^a^	49 (26.20%)	30 (25.64%)	19 (27.14%)	.821^a^
DS14 total	31.70 (12.15)	38.94 (8.24)	19.99 (7.39)	<.001^b^	31.87 (12.34)	39.19 (8.21)	19.65 (7.42)	<.001^b^
Negative affectivity	17.94 (6.59)	20.73 (4.77)	13.44 (6.65)	<.001^b^	17.90 (6.71)	20.89 (4.74)	12.89 (6.56)	<.001^b^
Social inhibition	13.76 (7.51)	18.21 (5.46)	6.56 (3.93)	<.001^b^	13.98 (7.49)	18.30 (5.50)	6.76 (3.97)	<.001^b^
PSC	16.89 (8.00)	17.67 (8.13)	15.63 (7.65)	.071^b^	16.84 (7.99)	17.58 (7.88)	15.60 (8.05)	.101^b^
GAD-7	11.78 (5.45)	13.00 (4.79)	9.83 (5.89)	<.001^b^	11.80 (5.42)	12.97 (4.76)	9.84 (5.90)	<.001^b^
PHQ-9	14.34 (6.10)	15.75 (5.67)	12.05 (6.11)	<.001^b^	14.24 (6.13)	15.69 (5.71)	11.80 (6.08)	<.001^b^

M, mean; SD, standard deviation; DS14, Type D Scale 14; PSC, Physical Symptom Checklist; GAD-7, Generalized Anxiety Disorder; PHQ-9, Patient Health Questionnaire.

PSC, GAD-7, and PHQ-9 are displayed as mean scores at intake.

a Pearson chi-square test.

b Students t test.

### Baseline Characteristics

**Table 1** shows the baseline characteristics for the SSRD patients for the total sample and for patients who completed the treatment. The prevalence of Type D personality in the total sample was 61.79% (*n* = 131). Type D patients did not differ significantly from non-Type D patients with respect to age, gender, and educational level. Compared to the non-Type D patients, patients with Type D personality experienced significantly higher levels of depression [*t* = 4.481, *p* < .001, mean difference 3.70, 95% CI: 2.07–5.33] and anxiety [*t* = 4.063, *p* < .001, mean difference 3.16, 95% CI: 1.62–4.69] at intake. Patients with Type D personality and without Type D personality did not differ significantly with regard to physical symptoms at baseline. A total of 81 patients (43.3%) had a chronic medical condition.

Further exploration of medical conditions showed that one patient was diagnosed with hypertension, eight were diagnosed with cardiovascular disease, one was diagnosed with rheumatoid arthritis, four were diagnosed with diabetes mellitus, and four were diagnosed with asthma/chronic obstructive pulmonary disease. With regard to physical comorbidity, 17 (9.1%) patients had no somatic disorder, 116 (62.0%) patients had one somatic disorder, 34 (18.2%) patients had two somatic disorders, 12 (6.4%) patients had three somatic disorders, and 8 (4.3%) patients had more than three somatic disorders.

With regard to the patients who completed treatment, the prevalence of Type D personality was 62.57% (*n* = 117). No significant differences were found regarding demographic variables between patients with and without a Type D personality who finished treatment. Compared to the non-Type D patients, patients with a Type D personality who finished treatment experienced significantly higher levels of depression [*t* = 4.404, *p* < .001, mean difference 3.89, 95% CI: 2.15–5.64 and anxiety [*t* = 3.757, *p* < .001, mean difference 3.12, 95% CI: 1.48–4.77 at intake. Demographic characteristics did not differ significantly between patients who completed treatment and the total sample of patients. Fourteen (56.0%) of the 25 patients who did not complete treatment had a Type D personality.

### Treatment Outcomes

#### Mean Changes From Baseline

The 187 patients who completed treatment showed a significant mean change of scores on the PSC (*M* = 16.77, *SD* = 7.80) and after treatment [(*M* = 13.43, *SD* = 9.66), *t* = 4.786, *p* < .001]. A significant mean change was also found between the mean scores on the GAD-7 at baseline (*M* = 11.73, *SD* = 5.24) and after treatment [(*M* = 9.02, *SD* = 6.40), *t* = 5.969, *p*< .001]. A significant mean change was also found between the PHQ-9 at baseline (*M* = 14.30, *SD* = 6.10) and after treatment [(*M* = 11.26, *SD* = 7.45), *t* = 5,758, *p* < .001]. ANOVA showed that scores for anxiety [*F* = 15.707, *p* < .001] and depression [*F* = 19.392, *p* < .001] were higher for patients with Type D personality compared to patients without Type D personality at baseline. Scores regarding physical symptoms did not differ significantly at baseline [*F* = 2.722, *p* = .101] but ANOVA with baseline measures as covariates showed that anxiety [*F* = 70.379, *p* < .001] and depression [*F* = 67.425, *p* < .001] scores at baseline explained these significant findings. **Table 2** shows the frequencies and percentages of patients who scored above the clinical cutoff on the PSC, GAD-7, and PHQ-9 before and after treatment. Results show that 93.5% had burdensome physical symptoms; 90.2% had clinical anxiety; and 96% of the patients were clinically depressed at intake. These percentages dropped significantly by 13.8% for physical symptoms, 21.1% for anxiety, and 20.0% for depression.

**Table 2:** Frequencies and percentages of patients who scored above/below cutoff at intake and at end of treatment.

Questionnaire		Intake assessment		End of treatment assessment		Change	McNemar test
		Below cutoff	Above cutoff	Below cutoff	Above cutoff		
	N	*n* (%)	*n* (%)	*n* (%)	*n* (%)		*p*
PSC	123	8 (6.5)	115 (93.5)	25 (20.3)	98 (79.7)	13.8%	<.001
GAD-7	123	12 (9.8)	111 (90.2)	38 (30.9)	85 (69.1)	21.1%	<.001
PHQ-9	125	5 (4.0)	120 (96.0)	30 (24.0)	95 (76.0)	20.0%	<.001

PSC, Physical Symptom Checklist; GAD-7, Generalized Anxiety Disorder; PHQ-9, Patient Health Questionnaire. Cutoff scores were 5 for each scale.

### Hierarchical Regression Analyses

#### Predicting Treatment Outcome From Type D Personality

##### Remission of Symptoms

**Table 3** shows the results of the logistic regression analyses for predicting remission and response from the dichotomous conceptualization of Type D personality. Type D personality had a significant negative effect on remission of anxiety [OR = .29, *p* = .009; Nagelkerke equaled 8.8%; χ^2^ = 6.931, *p* = .008], which was retained after controlling for baseline scores for anxiety [OR, .33, *p* = 0.39; Nagelkerke equaled 25.3%; χ^2^ = 22.732, *p* < .001]. Type D personality had a significant negative effect on remission of depression [OR = .21, *p* = .001; Nagelkerke equaled 12.9%; χ^2^ = 10.665, *p* = .001] but after we controlled for baseline scores for depression, this effect was not significant [OR = .36, *p* = .065; Nagelkerke equaled 24.1%; χ^2^ = 22.732, *p* < .001]. Type D personality was not associated with a remission of physical symptoms. These results suggest that the presence of Type D personality decreases the probability of a remission of anxiety and depression but not a remission of physical symptoms. When we controlled for baseline scores for the outcome of interest, the effect on remission of anxiety remained significant.

**Table 3:** Logistic regression predicting remission or response from Type D personality.

	Outcome variables								
	Physical symptoms (PSC)			Anxiety (GAD-7)			Depression (PHQ-9)		
	OR	95% CI	*ΔR* ^2d^	OR	95% CI	*ΔR* ^2d^	OR	95% CI	*ΔR* ^2d^
Results for remission									
Model 1^a^			.076			.009			.035
									
Model 2^b^			.044			.088*			.129*
Type D	.38	[.13; 1.10]		.29*	[.12; .73]		.21*	[.08; .55]	
Model 3^c^			.196			.253*			.241
Type D	.58	[.18; 1.87]		.33*			.36	[.13; 1.06]	
Results for response									
Model 1^a^			.010			.010			.002
									
Model 2^b^			.062*			.040			.032
Type D	.38*	[.17; .86]		.45	[.19; 1.07]		.49	[.22; 1.12]	
Mode 3^c^			.091			.067			.020
Type D	.44	[.19; 1.03]		.54	[.22; 1.30]		.58	[.25; 1.38]	

PSC, Physical Symptom Checklist; GAD-7, Generalized Anxiety Disorder; PHQ-9, Patient Health Questionnaire; OR, odds ratio; 95% CI, 95% confidence interval.

^a^Model 1 included background variables age, gender, and education level. ^b^Model 2 included the variables of Model 1 and added the dichotomous variable for Type D personality. ^c^Model 3 included the variables of Model 1 and Model 2 and added the baseline measurement for outcome of interest (PSC, GAD-7, or PHQ-9). ^d^Nagelkerke’s pseudo R^2^. All coefficients marked by an *are significant at the 5% significance level.

##### Treatment Response of Symptoms

Regarding response, the results show that Type D personality had a significant effect on response of physical symptoms [OR = .38, *p* = .021; Nagelkerke equaled 6.2%; χ^2^ = 5.396, *p* = .020], but after we controlled for baseline scores for physical symptoms, this effect was not significant [OR = .44, *p* = 0.59; Nagelkerke equaled 9.1%; χ^2^ = 8.298, *p* = .004]. Type D personality was not associated with a response of anxiety and depression. These results suggest that the presence of Type D personality decreases the probability of a response of physical symptoms but not a response of anxiety and depression. However, the significant effect of Type D personality on physical symptoms disappeared after the baseline scores on the PSC were controlled for.

#### Predicting Treatment Outcome on the Various Outcome Measures From NA, SI, and Their Interaction

**Table 4** shows the results of the logistic regression analyses for predicting remission and response from NA, SI, and NA × SI per outcome measure.

**Table 4:** Logistic regression predicting remission or response from Type D personality dimensions.

	Outcome variables								
	Physical symptoms (PSC)			Anxiety (GAD-7)			Depression (PHQ-9)		
	OR	95% CI	*ΔR* ^2e^	OR	95% CI	*ΔR* ^2e^	OR	95% CI	*ΔR* ^2e^
Results for remission									
Model 1^a^			.076			.009			.035
									
Model 2^b^			.164*			.173*			.154*
SI	1.03	[.94; 1.13]		1.01	[.94; 1.08]		.94	[.87; 1.02]	
NA	.85*	[.77; .94]		.85*	[.77; .94]		.91*	[.84; .99]	
Model 2^d^			.110			.155			.201
SI	1.01	[.91; 1.11]		1.00	[.93; 1.08]		.95	[.87; 1.04]	
NA	.90	[.81; 1.01]		.92	[.82; 1.02]		.99	[.90; 1.10]	
Model 3^c^			.062			.024			.003
SI	1.45	[1.00; 2.10]		1.22	[.92; 1.62]		1.00	[.79; 1.26]	
NA	1.07	[.83; 1.38]		.97	[.79; 1.18]		.94	[.81; 1.10]	
NA × SI	.98	[.96; 1.00]		.99	[.98; 1.00]		1.00	[.99; 1.01]	
Results for response									
Model 1^a^			.010			.010			.002
									
Model 2^b^			.109*			.113*			.058
SI	.98	[.92; 1.05]		1.00	[.94; 1.07]		.95	[.90; 1.02]	
NA	.91*	[.85; .98]		.89*	[.81; .97]		.97	[.90; 1.04]	
Model 2^d^			.125			.019			.008
SI	.98	[.91; 1.04]		1.00	[.94; 1.07]		.96	[.90; 1.02]	
NA	.94	[.87; 1.02]		.91	[.83; 1.01]		.98	[.91; 1.07]	
Model 3^c^			.009			.010			.003
SI	1.08	[.88; 1.33]		1.13	[.88; 1.44]		1.00	[.81; 1.21]	
NA	.97	[.84; 1.12]		.96	[.80; 1.14]		1.00	[.87; 1.13]	
NA × SI	1.00	[.99; 1.01]		.99	[.98; 1.01]		1.00	[.99; 1.01]	

PSC, Physical Symptom Checklist; GAD-7, Generalized Anxiety Disorder; PHQ-9, Patient Health Questionnaire; OR, odds ratio; 95% CI, 95% confidence interval; SI, social inhibition; NA, negative affectivity; NA × SI, interaction term of NA and SI.

^a^Model 1 included background variables age, gender, and education level. ^b^Model 2 included the variables of Model 1 and added the variables SI and NA. ^c^Model 3 included the variables of Model 1 and Model 2 and added the interaction variable NA × SI. ^d^Model 2^d^ included the variables of Model 1 and Model 2 and added the baseline measurement for outcome of interest (PSC, GAD-7, or PHQ-9). ^e^Nagelkerke’s pseudo R^2^. All coefficients marked by an *are significant at the 5% significance level.

##### Remission of Symptoms

The results for the remission (upper panel) of physical symptoms are as follows: NA had a significant effect on remission of physical symptoms [OR = .85, *p* = 002; and Nagelkerke equaled 16.4%; χ^2^ = 12.372, *p* = .002] The results for the remission of anxiety and depression followed the same trend: NA had a significant effect on the remission of anxiety [OR = .85, *p* = 001; Nagelkerke equaled 17.3%; χ^2^ = 14.029, *p* = .001], and NA had a significant effect on the remission of depression [OR = .91, *p* = .028; Nagelkerke equaled 15.4%; χ^2^ = 12.783, *p* = .002]. After we controlled for baseline scores, these effects were not significant for physical symptoms [OR = .90, *p* = .82; Nagelkerke equaled 11.0%; χ^2^ = 9.080, *p* = .003], for anxiety [OR = .92, *p* = .115; Nagelkerke equaled 15.5%; χ^2^ = 14.364, *p* < .001], and for depression [OR = 99, *p* = .890; Nagelkerke equaled 20.1%; χ^2^ = 19.057, *p* < .001]. SI did not show any significant effect on the remission of the outcome measures. These results suggest that if levels of NA are elevated, the probability of remission of physical symptoms, anxiety, and depression decreases, but this effect disappears when baseline scores are controlled for. NA × SI was not associated with the remission of physical symptoms, anxiety, or depression.

##### Treatment Response of Symptoms

The results for response (lower panel) showed that NA had a significant effect on response of physical symptoms [OR = .91, *p* = .016; Nagelkerke equaled 10.9%; χ^2^ = 9.580, *p* = .008]. NA also had a significant effect on the treatment response on anxiety [OR = .89, *p* = .007; Nagelkerke equaled 11.3%; χ^2^ = 9.626, *p* = .008]. After we controlled for baseline scores, these effects were not significant for physical symptoms [OR = .94, *p* = .125; Nagelkerke equaled 5.9%; χ^2^ = 5.571, *p* = .018] and for anxiety [OR = .91, *p* = .065; Nagelkerke equaled 1.9%; χ^2^ = 1.661, *p* = .198]. No significant associations were found regarding the response of depression. SI did not show any significant effects on the treatment responses for the outcome measures. These results suggest that if the levels of NA are elevated, the probability of response of physical symptoms and anxiety decreases. However, these effects disappeared when baseline scores were controlled for. NA × SI was not associated with a response of physical symptoms, anxiety, or depression.

## Discussion

### Key Results

This is the first study exploring the prevalence and association with treatment outcomes of Type D in patients with SSRD. The results show that the prevalence of Type D personality is 63% of the patients with SSRD who completed treatment, meaning that two out of three patients report a strong tendency to experience negative emotions and social inhibition. This prevalence exceeds the percentages reported in studies on Type D personality in various populations, including the general population (9), patients suffering from cardiovascular disease (6), and patients suffering from tinnitus (10), chronic pain (11), or fibromyalgia (12). All patients had fewer physical, anxious, and depressive symptoms at the end of treatment. However, after the correction for baseline anxiety and depression, the factor of having Type D personality significantly decreased only the effect on the remission of anxiety symptoms. NA and SI or NA × SI did not decrease the effect of the remission of physical symptoms, anxiety, or depression.

### Interpretation

This finding sheds new light on the association between Type D and anxiety and depression, as it confirms earlier reports of an association between the three but does not corroborate earlier findings that NA would be the only associated factor in Type D. Furthermore, this study still finds a negative effect on anxiety remission on both factors of Type D, which suggests that the main factor in Type D influencing treatment outcome in SSRD might be anxiety related. This would mean that the negative affectivity as well as the social inhibition would be anxiety related, not depression related, in Type D patients.

Earlier studies also reported an association not only between Type D personality and anxiety but also between SI and anxiety in the general population (41). Furthermore, the Type D components of NA and SI were also associated with anxiety (42, 43) and severity of anxiety (44) in a population of cardiac patients. These results suggest that anxiety may be an influencing factor with regard to treatment outcomes, and that this factor is worth studying in future research of patients with SSRD. The finding that Type D personality was not associated with treatment outcomes regarding physical symptoms in our study may be due to the flooring effect, as physical symptoms will not subside completely. This may be a case of the presence of chronic medical conditions. Our study did show that our sample consisted of patients with substantial physical diseases: 91% of the patients had at least one somatic disorder (e.g., rheumatoid arthritis, diabetes mellitus, and asthma), of which 10% had at least three somatic disorders.

### Strengths and Limitations

A strength of the study is that this is the first study to explore the influence of Type D personality as well as SI and NA and their interaction on treatment outcomes of patients suffering from SSRD. The limitations of the study are, firstly, that it is a non-experimental, observational design, which prevents causal interpretations. Hence, the results of this study should be interpreted with caution. Second, the subjects of this study were recruited in an outpatient SMHI in the Netherlands that is a Clinical Centre of Excellence for SSRD, which attracts patients with severe disorders. Furthermore, the treatment of patients with SSRD requires (22, 23) a standardized, tailored treatment approach that also prohibits a stratification for each kind of treatment that is provided at our center. Such stratification requires, if possible, a substantially large sample to preserve power. Nevertheless, this approach, which is in accordance with multidisciplinary guidelines (22, 23), can either consist of numerous combinations of ACT and/or CBT and/or PST sessions whether or not combined with a variety of pharmacological interventions, which renders the needed sample not feasible.

This is a longitudinal observational study that explores the association between Type D personality and treatment outcomes in patients with SSRD. All patients, both with and without Type D, received the same, standardized treatment, which consisted of modules of ACT, CBT, and PST, as well as of medication algorithms for pain, depression, or anxiety. These modules were tailored and delivered based on the patients’ needs and preferences, as well as on the progress of treatment over time as monitored with PROM. Hence, although this was a standardized approach, due to the tailoring, not all patients in the study received exactly the same treatment modules in the same sequence or containing all elements. This limitation has to be expected as this is not an experimental design, but an observational design, and an evaluation of the treatment modules themselves was not an objective of this study.

It is a limitation of the study that detailed information about medication use was not provided. Therefore, the influence of medication use as well as drug adherence on treatment outcome is unknown. This is an interesting subject for future studies. Furthermore, 43% of the patients in our sample were diagnosed with at least one chronic medical condition. The influence of these conditions with regard to treatment outcome was not explored, so caution should be exercised when interpreting our findings regarding patients with SSRD and chronic medical conditions. However, there was no significant association with Type D personality in patients who followed through on treatment, including drug treatment, and patients who did not, and only a small group did not follow through with treatment (*n* = 25, of which *n* = 14 were Type D). Hence, future research might explore if drug adherence might be influenced by NA or SI or by Type D personality in general. Nevertheless, it is worth exploring whether or not patients with SSRD, Type D personality, and, for instance, cardiovascular diseases benefit less from treatment compared to patients with SSRD, Type D personality, and other chronic medical conditions. Exploring the feasible negative effect of these cardiovascular diseases in patients with SSRD regarding treatment outcome should be the focus of future studies.

Finally, the results are not stratified for each kind of treatment that is provided at our center. Future studies should explore the possible difference remission/response of treatment for each kind of treatment offered to enable conclusions regarding which kind of treatment is most efficient regarding physical symptoms, anxiety, and depression. Also, treatment duration per kind of treatment (in days or hours) should also be included in future studies to evaluate the treatment duration of each specific kind of treatment and their effects on treatment outcomes. In addition, the effects of pharmacotherapy on symptom remission as well as the influence of known medical conditions are also worth exploring in this patient population.

#### Implications

The implications for clinical practice may be that more attention should be given to Type D with a specific focus on NA in diagnosis and treatment provision for patients with SSRD. At present, there are no well-evaluated evidence-based therapies yet that are specifically designed to alter the combination of NA and SI. Future research should evaluate whether patients with SSRD and Type D personality may benefit from interventions that address Type D personality and might improve the well-being and thus the functioning of this difficult-to-treat group of patients.

Treatment of patients with SSRD is challenging since these patients are complex (20) and may be burdened by clinical aspects, such as personality characteristics (such as Type D personality or alexithymia) (45) or neurocognitive impairment (46), which may interfere with treatment outcomes. These findings corroborate that the treatment of patients with SSRD can be influenced by multiple factors. Future studies should continue to explore personality factors and characteristics of patients with SSRD and explore the effects on treatment outcomes of these characteristics.

### Conclusions

The prevalence of Type D personality in patients with SSRD is 63%, which is higher than in other patient groups. Our results showed that patients had significantly fewer physical symptoms, anxiety, and depression after treatment. However, the presence of Type D personality only decreased the remission of anxiety, not of physical symptoms or of depression. Since Type D personality is associated with anxiety and severity of anxiety, future studies should explore to see if patients with SSRD and Type D personality may profit from interventions that include Type D personality.

## Ethics Statement

Patients were informed at intake about use of treatment outcome data for scientific research purposes on an anonymous basis. If the patient refused to give her consent, this was recorded in the administration system and the patient was excluded from the study. Data of all patients who participated in the study were anonymized to ensure privacy. For this study, we used a selection of the ROM questionnaires assessed at baseline and at the end of treatment. Patients could decide to withdraw from the study at any time without any consequences for their treatment. The scientific committee of GGZ Breburg approved of this study (file number: CWO 2014-11).

## Author Contributions

LV: contributed to writing the manuscript, doing analyses, interpreting results. EH: contributed to editing the manuscript, doing analyses, interpreting results. ET: contributed to obtaining the data, writing the concept manuscript. KB: contributed to editing the manuscript, supervising analyses. JES: contributed to editing the manuscript. CF-C: supervisor in the project, designed and facilitated the study, supervised the analyses, edited the manuscript and approved the final version.

## Conflict of Interest Statement

The authors declare that the research was conducted in the absence of any commercial or financial relationships that could be construed as a potential conflict of interest.
